# Modelling of ankle joint range of motion and landing quality scores in female soccer players with quantile regression approach

**DOI:** 10.1371/journal.pone.0325180

**Published:** 2025-06-05

**Authors:** Niloofar Fakhraei Rad, Mohammad Alimoradi, Bogdan Antohe, Hüseyin Şahin Uysal, Sezgin Korkmaz, Nicola Relph, Zahra Mohammadian

**Affiliations:** 1 School of Health and Rehabilitation Sciences, Physiotherapy, The University of Queensland, Brisbane, Queensland, Australia; 2 Faculty of Sports Sciences, Shahid Bahonar University of Kerman, Kerman, Iran; 3 ”Vasile Alecsandri” University of Bacău, Bacău, România; 4 Burdur Mehmet Akif Ersoy University, Faculty of Sport Sciences, Burdur, Turkey; 5 Edge Hill University, Health Research Institute, Faculty of Health, Social Care and Medicine, Ormskirk, United Kingdom; 6 Faculty of Physical Education and Sport Sciences, University of Tehran, Tehran, Iran; Università degli Studi di Milano: Universita degli Studi di Milano, ITALY

## Abstract

**Purpose:**

Ankle dorsiflexion range of motion (DF-ROM) has been shown to be associated with poor landing posture. This study aimed to model the relationship between DF-ROM and landing quality during the Soccer-Specific Jump-Landing task (SSJL) on the non-dominant extremity in elite and amateur female soccer players using a quantile regression approach.

**Methods:**

55 amateur and 47 professional female soccer players participated in the study. The relationship between DF-ROM and SSJL quality was evaluated using Pearson’s product-moment correlation analysis and Quantile regression modelling.

**Results:**

There was a statistically significant negative correlation between DF-ROM and SSJL landing quality among female soccer players (*r* = − 0.33, *p* = 0.001). Moreover, there was a statistically significant negative correlation between DF-ROM and SSJL landing quality in amateur female soccer players (*r* = − 0.63, *p* = 0.001), no significant correlation was found in elite female soccer players (*r* = 0.22, *p* = 0.13). Quantile regression results for amateur female soccer players indicate that the association between ankle DF-ROM and landing quality is stronger in athletes with higher SSJL landing quality scores (e.g., Q75 and Q90) compared to those with lower scores (e.g., Q10 and Q25). This suggests that ankle DF-ROM plays a more critical role in achieving higher-quality landing techniques than in poorer techniques.

**Conclusions:**

Higher quality landing techniques in female soccer players are associated with larger ranges of ankle DF-ROM. Therefore, ankle DF-ROM level may be a functional clinical measurement for amateur female athletes in determining landing-related injury risk factors during SSJL.

## Introduction

Soccer is widely recognized as a prevalent sport worldwide and is increasingly attractive to female participation [1]. Research has shown that female soccer has experienced rapid growth, encompassing professional, semi-professional, and amateur levels, and is considered the fastest-growing sport globally [2]. Since soccer involves high-intensity technical tasks such as shooting, passing, dribbling, jumping, acceleration, deceleration, and stopping, the increase in female participation has been accompanied by a rise in injury rates [3]. While shoulder and head/neck injuries do occur, 86.8% of soccer-related injuries originate from lower extremity joints, such as the ankle and knee [4]. The ankle, the lowest component of the kinetic chain in the lower limb, is commonly affected by sprains, Achilles tendon injuries, and plantar fascia injuries during competition [5]. These injuries are likely to limit future ankle range of motion [6], which has been identified as a significant risk factor for other injuries such as anterior cruciate ligament (ACL) injury [7].

Since 82% of ACL injury cases in soccer occur in non-contact situations [8], researchers have developed several testing protocols conducted in a non-contact environment to identify players at risk of injury [9]. One of these protocols is the landing technique score [10]. Limited ankle dorsiflexion range of motion (DF-ROM) can affect landing technique by altering joint loading, forcing compensatory movements such as increased foot pronation, reduced knee sagittal excuration and greater hip stiffness, leading to suboptimal postures like flat-footed landings, and reducing overall balance and stability, thereby increasing the risk of injury [7,11]. Also the relationship between increased stress on the knee joint during landing and the risk of ACL injury underscores the importance of evidence-based strategies to mitigate such risks. Therefore, landing technique testing is often used in an attempt to predict anterior cruciate ligament injuries in soccer [12]. Although various protocols have recommended landing techniques, a generallanding test protocol may not be suitable for interpreting sport-specific mechanical actions. For example, soccer players can focus on multiple tasks during a jumping motion, including both ball tracking and landing mechanics. Furthermore, current landing technique tests may not be sufficient to interpret sport-specific landing quality [13]. The relationship between DF-ROM and landing technique could be better diagnosed with the soccer-specific jump-landing task (SSJL). Indeed, previous research has reported a moderate negative correlation (*r* = − 0.450, *p* = 0.006) between DF-ROM levels and SSJL in male soccer players [13], suggesting that lower DF-ROM may contribute to a higher risk of landing errors. Female athletes exhibit greater knee valgus angles and reduced neuromuscular control during landing tasks compared to males, which may exacerbate the effects of limited ankle DF-ROM on landing mechanics [12,14,15]. Therefore, it is critical to investigate whether this relationship holds true in female athletes to inform tailored injury prevention strategies.

When comparing the rate of anterior cruciate ligament injuries between genders, a study by researchers found that female soccer players were 5.36 times more likely to experience an acute injury [16]. These results emphasized the importance of optimal ankle joint range of motion for female soccer players, and the existence of mechanical differences between genders. Although the relationship between DF-ROM and SSJL has been evaluated in male soccer players [13], the relationship between DF-ROM levels and soccer-specific landing quality in female soccer players has not been investigated. Furthermore, evaluating the current subject area with traditional correlation tests may limit interpretation of the relationship between DF-ROM and SSJL in elite and amateur female soccer players. Standard correlation tests are based on average values in a data set, and may fail to capture non-linear relationships between variables. Quantile regression allows for a nuanced analysis of how DF-ROM impacts athletes at different skill levels (e.g., those with high vs. low landing error scores), which linear models may overlook [17].

This study focused on the non-dominant ankle DF-ROM, as ACL injuries are more frequently associated with unilateral loading on the non-dominant lower limb in females. Research has shown that the knee valgus angle at initial contact, a critical biomechanical risk factor for ACL injuries is significantly greater in the non-dominant leg (0.8° ± 5.2°) compared to the dominant leg (−0.9° ± 4.9°) [18,19].

This suggests that the non-dominant leg may be more susceptible to landing errors and associated injuries, justifying its targeted evaluation in this study. By focusing on the non-dominant leg, this study aimed to model the relationship between non-dominant ankle DF-ROM and landing quality during the SSJL task in elite and amateur female soccer players using a quantile regression approach.

## Methods

### Study design

The study design was a single-blind observational, cross-sectional study design. Data were analyzed by a researcher blinded to participant characteristics and not involved in the data collection process. The Quality Output Checklist and Content Assessment (QuOCCA) checklist was used to enhance the methodological quality of the study [20], and it is presented in Appendix 2. Additionally, the study protocol was pre-registered on the Open Science Framework (OSF) (https://doi.org/10.17605/OSF.IO/KP7XA, accessed date: 11.12.2023), and details of the study files are available on the OSF website (https://osf.io/mtney/ accessed date: 11.12.2023).

### Participants

102 female soccer players (age: 21.30 ± 2.14 years, height: 165.10 ± 6.05 cm, weight: 56.56 ± 3.97 kg, BMI: 20.91 ± 2.25 kg ∙ m^2^) from Iran’s Shahin and Khatoon soccer clubs were recruited for the study between 18/12/2023–25/12/2023. The participants consisted of 55 amateur and 47 elite-level female soccer players. The participants were selected based on the following criteria in the study: (i) being over 18 years of age, (ii) having at least three years of experience in the field for amateur players, (iii) having competed in the premier, first, or second leagues for professional players (iv) participating in at least two training sessions per week, (v) not having suffered any severe lower limb injuries in the past year (such as ankle or knee sprains, or ACL tears), (vi) being outside the menstrual phase [21].

The study sample was determined using the simple random method, and the sample size was determined based on the results of a previous study [13]. A *priori* power analysis was performed using G*Power software (version 3.1, University of Dusseldorf, Germany) based on the following reference values: α = 0.05, β = 0.80, *r* = 0.35, two-tailed, and a bivariate normal model test. The results showed that the minimum sample size should be 61 participants, while the study included 102 female soccer players. This study was approved by the Vasile Alecsandri University Ethics Committee (decision no: 42/2, 14.12.2023) and was conducted in accordance with the Declaration of Helsinki. All participants provided written informed consent before being included.

### Experimental procedure

This study was conducted in Kerman, Iran and measurements were performed on the natural grass fields at the Kargar and Fajr stadiums. A track and field coach with seven years of experience in sports carried out the study protocol. Since the study protocol included jumping and landing tasks that required a high biomechanical load, a standardized 15-minute warm-up protocol was implemented to prevent injuries to the athletes. This warm-up protocol consisted of a 7-minute jog, double-leg squats (2 sets x 10 reps), and dynamic stretches for the hamstrings, quadriceps, and calves (2 sets x 10 reps). Before commencing the measurements, the device calibrations were verified, and all participants underwent the tests while wearing identical attire. Then non-dominant limb test, weight-bearing lunge test, vertical jump test, and soccer-specific jump landing test were performed, respectively. The best test results of the soccer players were used for statistical analysis.

### Meavsurements

#### Non-dominant limb test.

The non-dominant leg of female soccer players was assessed to measure the DF-ROM level, as it exhibits reduced proprioceptive acuity and strength compared to the dominant limb, increasing its vulnerability to landing errors [22,23].The ball strike test was used to determine the dominant foot. This test was carried out according to the instructions specified by the researchers, and female soccer players kicked the ball into a target located four meters away. The leg used when hitting the target was considered the dominant leg. Researchers reported 100% agreement between the dominant leg reported by participants and the leg used during the ball-striking test [24].

#### Weight−bearing lunge test.

**Fig 1:**
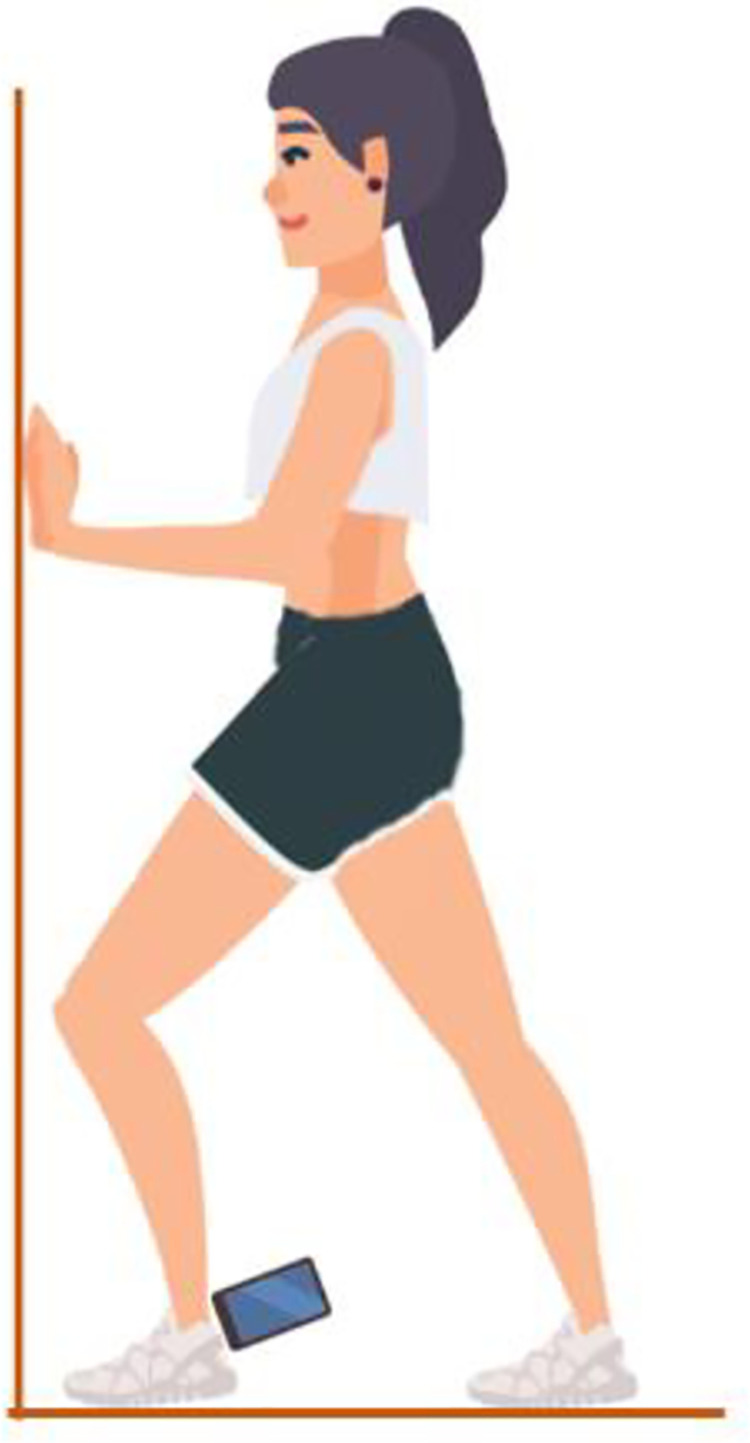
Measurement of ankle dorsi flexion joint range of motion with Inclinometer & Bubble Level mobile app.

The weight−bearing lunge test was used in the study to determine the DF-ROM level of female soccer players. Researchers stated that the test has high validity (*r* = 0.71) and reliability (ICC = 0.80 to 0.99) for evaluating DF-ROM [25,26]. This test was performed using the Inclinometer & Bubble Level (InclineSense Technologies, Iran) mobile app, which has high validity and reliability [27]. Measurements were performed according to instructions in previous study protocol [28]. Before measurement, each device was placed with its long axis on the ground and calibrated to 0° next to the participant. The closed kinetic chain test, which simulates ankle and knee joint loading during the landing mission and provides complete DF-ROM measurement, was chosen to perform the measurement. Participants were asked to place their hands shoulder-width apart on the wall. The non-dominant leg was placed 10 cm from the wall, with the knee in line with the second toe. The dominant leg was placed behind the non-dominant leg with the knee extended. A mobile phone was placed on the flat surface behind the Achilles tendon, approximately one centimeter above the posterior calcaneal tuberosity and perpendicular to the tibia. An inclinometer application called Inclinometer & Bubble Level (or BublePro App) was installed on a mobile telephone to measure ankle DF-ROM. The subjects continued the test by flexing the knee until the heel lifted off the ground. DF-ROM measurement was completed before the heel was lifted off the ground. The test was repeated three times for the non-dominant leg, and the maximum value was used for data analysis. The details of the test protocol are presented in Fig 1.

#### Vertical jump test.

The vertical jump height of female soccer players was determined with a traditional vertical jump test, The vertical jump was measured by having each participant stand beside a wall with their non-dominant side. With feet firmly planted on the ground and hips positioned as close as possible to the wall, the participant extended the arm nearest the wall and reached as high as possible. This point of maximum reach was then marked on the wall. Throughout this exercise, it was essential to ensure that no part of the feet lost contact with the ground [29]. After marking the wall, the players jumped as high as possible. The wall was marked again at the highest point reached by the participants. The difference between these two measurements was recorded as the participant’s maximum vertical jump. Participants were allowed to perform a maximum of three jumps, and the height of the ball was determined by taking the average of the two highest measurements.

#### Soccer-specific jump landing test (SSJL).

The SSJL test, an adapted version of the Landing Error Scoring System (LESS), was utilized to assess the landing biomechanics of soccer players. The reliability of the test was confirmed previously [30]. Data was collected as per previous protocols to increase the validity and reliability of the data collected [31,32]. The test was recorded with an external video.

**Fig 2:**
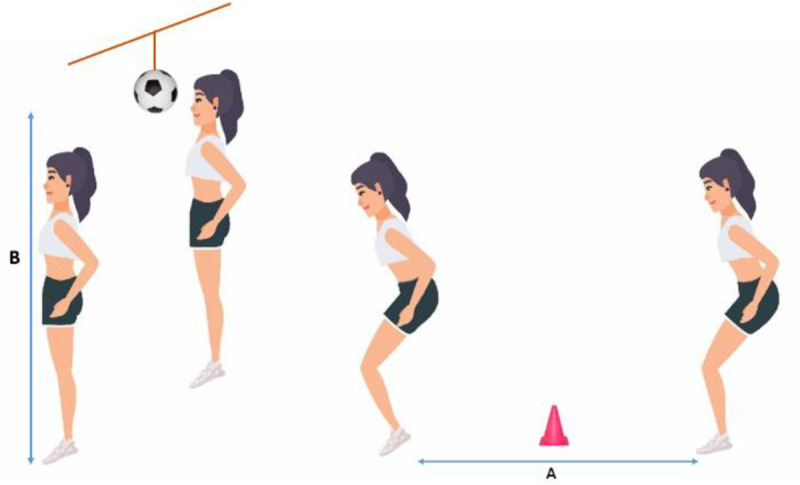
Soccer-Specific Jump-landing task test. A: 50% of the participants’ height from the landing site to the start position; B: Centre of the soccer ball is 50% of the participants’ maximum vertical jump height.

To perform the test, participants had to jump over a 7.5 − centimeter cone with the landing point positioned at a distance equal to half their height. Participants were instructed to jump vertically after landing on both feet and head a soccer ball suspended at a point equivalent to half of their maximum vertical jump height, landing approximately at the same location [33]. Additionally, the test was performed without shoes since footwear can impact jumping performance. To record the tests, two digital video cameras (GoPro HERO 9, USA) were positioned 3 meters in front and to the right of the participants [13]. The test was performed three times, and the best result was used for data analysis. Each participant’s landing technique was evaluated using Kinovea (version 0.7.10, USA), a valid and reliable software [34]. The evaluation was based on the criteria outlined in Appendix 1, adapted from the Landing Error Scoring System (LESS) [35]. The LESS test was administered to evaluate each participant’s landing technique and assessed 17 items on a scale ranging from 0 to 19. A higher score, indicating more landing errors, suggested potentially high−risk movement patterns. All participants were evaluated by an assessor trained in the scoring protocol. Movements of the lower extremities and trunk were analysed at two specific moments: (i) between the first contact of the foot with the ground and (ii) between the first contact with the land and maximum knee flexion. Because several studies suggest that the non-dominant extremity is more susceptible to injury than the dominant extremity during landing strategies in female athletes [18,19], the evaluation was conducted solely on the non−dominant extremity. The details of the test protocol are presented in Fig 2.

### Statistical analysis

The study evaluated the relationship between DF-ROM and SSJL of elite and amateur female soccer players. The Kolmogorov-Smirnov test was used to check the assumption of normality, which was confirmed. The relationship between variables was assessed using Pearson’s product-moment correlation analysis. The correlation coefficient is interpreted based on the following references [36]: insignificant (< 0.10), small (0.10 − 0.29), moderate (0.30 − 0.49), strong (0.50 − 0.69), very strong (0.70 − 0.89), or excellent (> 0.90).

**Fig 3:**
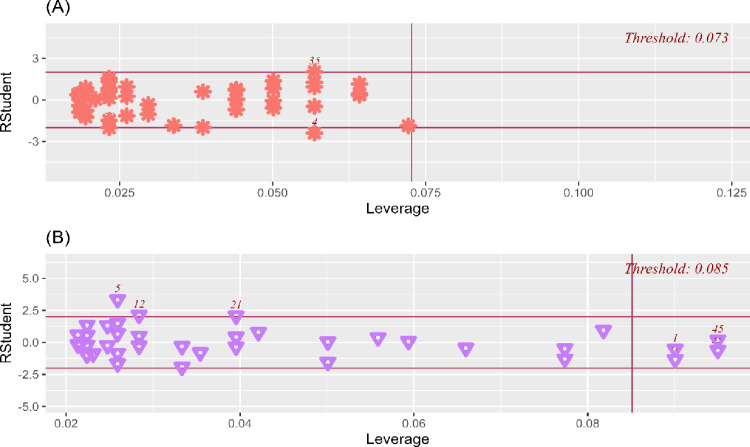
The results of outlier analysis of amateur and elite female football player groups. **A:** Amateur female football players group; **B:** Elite female football players group.

In addition to correlation analysis, regression analyses were also performed. While potential outliers in the study data were analysed using Cook’s distance, heteroskedasticity was assessed using the Breusch-Pagan test [37]. Outliers were identified in the study data (Fig 3), heteroscedasticity was also observed (*p* < 0.05). Therefore, the Akaike Information Criterion (AIC) test was performed to determine the most appropriate method for the regression analysis in the study. A lower AIC value indicates a more appropriate regression analysis method [38]. Linear regression analysis was compared with quantile regression (QR) analysis to determine whether DF-ROM is a predictor of SSJL. Details regarding the comparison results are presented in Table 1.

**Table 1:** Comparison results of linear and quantile regression analyses based on Akaike’s Information Criterion.

𝜏	Amateur Female Soccer Players					Elite Female Soccer Players			
	LR		QR		LR			QR	
	df	AIC	df	AIC	Df		AIC	df	AIC
0.10	3	200.95*	2	215.62	3		162.65*	2	165.78
0.25	3	200.95*	2	219.73	3		162.65	2	160.03*
0.50	3	200.95*	2	202.75	3		162.65	2	161.57*
0.75	3	200.95	2	194.17*	3		162.65*	2	171.44
0.90	3	200.95	2	196.71*	3		162.65*	2	196.15

***Legend*.** *df*: Freedom of degrees; AIC: Akaike’s Information Criterion; *: It is represents a more appropriate method to analyse data; 𝜏: Percentile of data (i.e., 0.90 represents the 10% that contains the highest landing scores in the dataset.); LR: Linear regression; QR: Quantile regression.

The study data included outliers and revealed heteroscedasticity. Additionally, the AIC results showed conflicting outcomes for different percentiles. Therefore, both linear regression and QR analyses were performed in the study. While evaluating the relationship between two variables in a data set, QR analysis is based on the median values instead of the mean values [39]. In the study, QR analysis was calculated using the following equation:


$$Y\;=\;\beta_{0}(q)\;+\;\beta_{1}(q)\;*\;X_{1}\;+\;\beta_{2}(q)\;*\;X_{2}\;+\;\ldots \;+\;\beta_{r}(q)\;*\;X_{r}\;+\;\varepsilon .$$

Y represents the dependent variable in this equation, while β defines the estimation coefficients. The expression x represents the independent variable, and q represents the percentiles. Finally, the symbol ε represents the error term in the equation. QR analysis was performed based on previous studies, and five percentiles (10%, 25%, 50%, 75%, 90%) were selected [40]. While the Q10 and Q25 percentiles included female soccer players with lower SSJL landing quality scores, the Q75 and Q90 percentiles included female soccer players with higher SSJL landing quality scores.

R (ver. 4.2.2., Core Team, Vienna, Austuria) software was used for the statistical analysis and data visualization of this study. These operations were performed using the {quantreg}, {olsrr}, {ggplot2}, {gridExtra}, {patchwork}, {gtsummary}, and {performance} packages. Statistical significance was set at *p* < 0.05 for all analyses. The R codes of the study are presented in Appendix 3.

## Results

One hundred two female soccer players were included in the study, and all of them participated in the test session. No injuries or incidents occurred during the testing session following the study protocol. The mean DF-ROM, measured using the weight-bearing lunge test, was 35.00° ± 7.62° (range: 22.48° to 46.56°) in amateur female soccer players and 33.60° ± 5.74° (range: 22.84° to 44.19°) in elite female soccer players. While the amateur group’s mean falls within the normative range of 35°- 45° reported for healthy adults during weight-bearing activities, the elite group’s mean is slightly below this range, although individual values still largely overlap with the normative spectrum [41].

**Fig 4:**
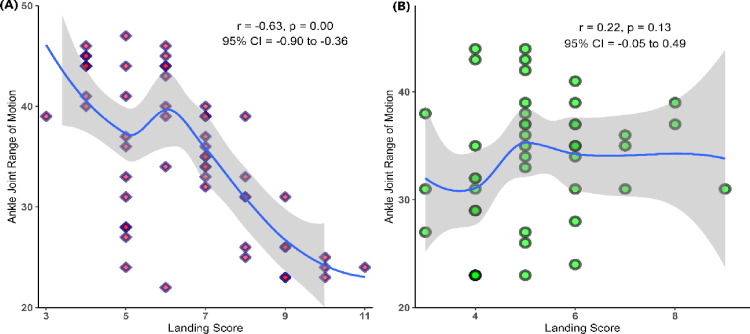
Results of the relationship between football-specific jump landing scores and ankle joint range of motion of female football players. **A:** Amateur female football players group; **B:** Elite female football players group.

There was a significant negative correlation between DF-ROM and SSJL landing quality among female soccer players (*r* = − 0.33, *p* = 0.001). A strong negative correlation was found between SSJL and DF-ROM of amateur female soccer players (*r* = − 0.63, *p* = 0.00). However, there was no statistically significant relationship between SSJL and DF-ROM in elite female soccer players (*r* = 0.22, *p* = 0.13). Details of the correlation analyses are presented in Fig 4.

While linear regression analyses reported that DF-ROM of amateur female soccer players could be a predictor for SSJL (β = − 0.16, *p* < 0001), these results were not observed in elite female athletes (β = 0.04, p = 0.20). QR analyses reported that DF-ROM levels of elite female soccer players in different percentiles were not a predictor of SSJL. Similarly, it was revealed that the DF-ROM level of amateur female soccer players in the Q10 percentile did not affect SSJL (β = − 0.06, *p* = 0.08). However, it was revealed that DF-ROM could be a more critical predictor as SSJL errors increased. The strongest negative relationship was detected between the DF-ROM level and SSJL errors of amateur female soccer players in the Q90 percentile with the highest SSJL scores (β = − 0.20, *p* < 0.001). Details of the regression analyses are presented in Table 2.

**Table 2:** Linear and quantile regression analysis results based on the relationship between ankle joint range of motion and soccer-specific jump landing score.

Analyses/Group	AFSP			EFSP		
	β	95% CI	p-value	β	95% CI	p-value
LR	−0.16	−0.21 to −0.11	<0.001*	0.04	−0.02, to 0.11	0.2
QR10	−0.06	−0.13 to 0.01	0.08	0.00	−0.08 to 0.08	>0.9
QR25	−0.09	−0.17 to 0.00	0.03*	0.07	−0.03 to 0.16	0.2
QR50	−0.18	−0.27 to −0.09	<0.001*	0.00	−0.08 to 0.08	>0.9
QR75	−0.17	−0.21 to −0.12	<0.001*	0.00	−0.12 to 0.12	>0.9
QR90	−0.20	−0.26 to −0.14	<0.001*	0.09	−0.12 to 0.30	0.4

***Legend.*** AFSP: Amateur female soccer players; EFSP: Elite female soccer players; LR: Linear regression; QR: Quantile regression; 95% CI: Upper and lower limit of %95 confidence interval.

## Discussion

This study assessed the correlation between non-dominant ankle DF-ROM and landing quality during the SSJL task in female elite and amateur soccer players and model the results using a quantile regression approach. While the results of the correlational analysis indicated a strong negative correlation between landing errors and ankle DF-ROM level in amateur female soccer players (*r* = − 0.63, 95%CI = − 0.36 to − 0.90, *p* = 0.00), the analysis results for elite female soccer players were not statistically significant (*r* = 0.22, 95%CI = − 0.05 to 0.49, *p* = 0.13). Quantile regression analysis results for amateur female soccer players showed that ankle DF-ROM level affected landing quality more in the group with high landing errors (i.e., Q75 and Q90) compared to the group with low landing error scores (i.e., Q10 and Q25). While these results suggest that the DF-ROM level would not affect the landing quality in elite female soccer players, they revealed that the DF-ROM level was more significant for amateur female soccer players with a high landing quality score.

Our findings were consistent with a previous study on male soccer players using a similar protocol [13]. However, the correlation level was higher in amateur female soccer players than their male counterparts. The results of the current study for amateur female soccer players provide further supporting evidence thats biomechanical indicators and injury rates differ between genders [16]. Conversely, the lack of a significant correlation between the DF-ROM level of elite female soccer players and the quality of landing during SSJL may be attributed to various factors. For example, elite athletes have a lower injury rate than amateur athletes [42], exhibit better attention and decision-making processes [43], and achieve higher sports performance outcomes [44]. This may explain the statistically non-significant results for elite soccer players.

Several physiological factors may contribute to the statistically significant correlation between DF-ROM and the landing quality of female amateur soccer players. Due to limited ankle DF-ROM, athletes may be unable to absorb the ground reaction forces during landing in all jumping activities [45,46]. Additionally, limitations in range of motion may increase sagittal and medial displacements of the knee, predisposing athletes to ACL injury [28]. If the knee joint cannot compensate for the ankle DF-ROM deficiency, the body may adopt the hip strategy as a compensatory kinematic chain movement strategy [47]. If the lower extremity is unable to absorb the forces generated during landing, the trunk will compensate for these deviations, resulting in changes in whole-body movement patterns [11]. Limited ankle DF-ROM can impact multiple joints and their corresponding movements, resulting in compromised movement patterns that may elevate the risk of injury [45]. The mechanism behind the impact of limited ankle DF-ROM on the landing quality of amateur female soccer players can be explained by this hypothesis.

Additionally, the absence of a correlation between DF-ROM and dorsiflexion displacement may result in variations in landing technique among female amateur soccer players. These differences in landing styles may affect landing biomechanics, particularly dorsiflexion displacement, independently of passive dorsiflexion ROM [48]. Researchers reported that limb dominance can significantly affect the coordination of skilled movement patterns, and biomechanical symmetry between the dominant and non-dominant limbs is crucial for competent performance [49]. Researchers also observed that the non-dominant limb is more vulnerable to injury due to inadequate stability [22,50]. A study analysing asymmetries in leg dominance during the landing test showed that lower extremity biomechanics, specifically knee and hip joint range of motion, were significantly lower in the non-dominant leg [22]. These findings may explain why female amateur soccer players have poorer movement patterns for their non-dominant limbs.

There are some limitations in the study. The first limitation concerns performing the SSJL task on the non-dominant extremity. Tests on the dominant extremity may yield varying results for female amateur soccer players. Future studies should control for covariates such as weekly training volume and prior injury to isolate the impact of DF-ROM on landing mechanics. Moreover, studying the effects of various joints during SSJL could be a research topic that would yield more robust empirical evidence.

In conclusion, there appears to be no relationship between landing quality and DF-ROM levels of elite female soccer players during the SSJL test, likely due to elite environments that include conditioning training.. However, the landing quality of female amateur soccer players during the SSJL varies depending on the DF-ROM level. Limited ankle DF-ROM affects the landing quality score of all amateur female soccer players. However, the effect of DF-ROM on landing quality scores increases in amateur female soccer players who already have high landing quality scores. These findings have important implications for the growing sport of women’s soccer and injury prevention. For amateur players, integrating dynamic stretching (e.g., weighted lunges) and proprioceptive training may enhance DF-ROM and reduce compensatory movements during landing. Such interventions could improve landing quality and potentially reduce the risk of lower limb injuries, especially for those with already high landing quality scores. As the sport continues to develop, integrating ankle mobility and flexibility training into conditioning programs could help optimize performance and mitigate injury risks, particularly for players in the amateur ranks.

## Supporting information

S1 File Appendix File. (DOCX)
