# Supplementary material for: Modelling of ankle joint range of motion and landing quality scores in female soccer players with quantile regression approach
Source: PLoS One. 2025 Jun 5;20(6):e0325180. doi: 10.1371/journal.pone.0325180 (PMC12140249; doi:10.1371/journal.pone.0325180)
Supplement: S1 File — (DOCX) [file pone.0325180.s001.docx]

**Appendix 1.** LESS items.

| **LESS items** | **Operational definition** | **Camera view** | **LESS score** |
| --- | --- | --- | --- |
| 1. Knee flexion angle at initial contact | At the time point of initial contact, if the knee of the test leg is flexed more than 30 degrees, score YES. If the knee is not flexed more than 30 degrees, score NO. | Sagittal | Yes = 0, No = 1 |
| 2. Hip flexion angle at initial contact | At the time point of initial contact, if the thigh of the test leg is in line with the trunk then the hips are not flexed and score NO. If the thigh of the test leg is flexed on the trunk, score YES. | Sagittal | Yes = 0, No = 1 |
| 3. Trunk flexion angle at initial contact | At the time point of initial contact, if the trunk is vertical or extended on the hips, score NO. If the trunk is flexed on the hips, score YES. | Sagittal | Yes = 0, No = 1 |
| 4. Ankle plantarflexion angle at initial contact | If the foot of the test leg lands toe to heel, score YES. If the foot of the test leg lands heel to toe or with a flat foot, score NO. | Sagittal | Yes = 0, No = 1 |
| 5. Knee valgus angle at initial contact | At the time point of initial contact, draw a line straight down from the center of the patella. If the line goes through the midfoot, score NO. If the line is medial to the midfoot, score YES. | Frontal | Yes = 0, No = 1 |
| 6. Lateral trunk flexion angle at initial contact | At the time point of initial contact, if the midline of the trunk is flexed to the left or the right side of the body, score YES. If the trunk is not flexed to the left or right side of the body, score NO. | Frontal | Yes = 1, No = 0 |
| 7. Stance width–Wide | Once the entire foot is in contact with the ground, draw a line down from the tip of the shoulders. If the line on the side of the test leg is inside the foot of the test leg then greater than shoulder width (wide), score YES. If the test foot is internally or externally rotated, grade the stance width based on heel placement. | Frontal | Yes = 1, No = 0 |
| 8. Stance width–Narrow | Once the entire foot is in contact with the ground, draw a line down from the tip of the shoulders. If the line on the side of the test leg is outside of the foot then score less than shoulder width (narrow), score YES. If the test foot is internally or externally rotated, grade the stance width based on heel placement. | Frontal | Yes = 1, No = 0 |
| 9. Foot position–Toe in | If the foot of the test leg is internally more than 30 degrees between the time period of initial contact and max knee flexion, then score YES. If the foot is not internally rotated more than 30 degrees between the time period of initial contact to max knee flexion, score NO. | Frontal | Yes = 1, No = 0 |
| 10. Foot position–Toe out | If the foot of the test leg is externally rotated more than 30 degrees between the time period of initial contact and max knee flexion, then score YES. If the foot is not externally rotated more than 30 degrees between the time period of initial contact to max knee flexion, score NO. | Frontal | Yes = 1, No = 0 |
| 11. Symmetric initial foot contact | If one foot lands before the other or if one foot lands heel to toe and the other lands toe to heel, score NO. If the feet land symmetrically, score YES. | Frontal | Yes = 0, No = 1 |
| 12. Knee flexion displacement | If the knee of the test leg flexes more than 45 degrees from initial contact to max knee flexion, score YES. If the knee of the test leg does not flex more than 45 degrees, score NO. | Sagittal | Yes = 0, No = 1 |
| 13. Hip flexion at max knee flexion | If the thigh of the test leg flexes more on the trunk from initial contact to max knee flexion angle, score YES. | Sagittal | Yes = 0, No = 1 |
| 14. Trunk flexion at max knee flexion | If the trunk flexes more from the point of initial contact to max knee flexion, score YES. If the trunk does not flex more, score NO. | Sagittal | Yes = 0, No = 1 |
| 15. Knee valgus displacement | At the point of max knee valgus on the test leg, draw a line straight down from the center of the patella. If the line runs through the great toe or is medial to the great toe, score YES. If the line is lateral to the great toe, score NO. | Frontal | Yes = 1, No = 0 |
| 16. Joint displacement | Watch the sagittal plane motion at the hips and knees from initial contact to max knee flexion angle. If the participant goes through large displacement of the trunk, hips, and knees, then score SOFT. If the participant goes through some trunk, hip, and knee displacement but not a large amount, then AVERAGE. If the participant goes through very little, if any trunk, hip, and knee displacement, then STIFF. | Sagittal | Soft = 0, Av. = 1, Stiff = 2 |
| 17. Overall impression | Score EXCELLENT if the participant displays a soft landing and no frontal plane motion at the knee, Score POOR if the participant displays a stiff landing and large frontal plane motion at the knee. All other landings, score AVERAGE. | Sagittal and Frontal | Ex. = 0, Av. = 1, Poor = 2 |

***Legend:*** LESS: landing error scoring system; Av: average; Ex: excellent.

Sutton, B. (2022). Understanding the modified landing error scoring system (LESS test). Accesed date: 05.12.2023. <https://blog.nasm.org/sports-performance/modified-landing-error-scoring-system-less-acl-injury>

**Appendix 2.** QuOCCA (Quality Output Checklist and Content Assessment) checklist of the study

| **QuOCCA**  **Quality Output Checklist and Content Assessment** | | | | |
| --- | --- | --- | --- | --- |
| This checklist is intended for peer-reviewed research papers. It should not be used for reviews, chapters, editorials etc. | | | | |
| **Manuscript Title:** | Modeling of Ankle Joint Range of Motion and Landing Quality Scores in Female Soccer Players with Quantile Regression Approach | | | |
| **Manuscript Authors:** | Niloofar Fakhraei Rad, Mohammad Alimoradi, Bogdan Antohe, Hüseyin Şahin Uysal, Sezgin Korkmaz, Zahra Mohammadian | | | |
| **Person submitting this form:** | Bogdan Antohe, Hüseyin Şahin Uysal | | | |
| **Date:** | 11.12.2023 | | | |
| TRANSPARENCY: | | N/A | YES | NO |
| 1a.  1b. | Were the study’s hypotheses and analyses plans registered prior to the conduct of the study (i.e. pre-registered)?  If so, was the main conclusion reported in the abstract (or summary) based on the primary hypothesis /outcome? |  | 🗸  Line 116 |  |
|  |  |  | 🗸  Line 33 |  |
| 2. | Are the primary data accessible to independent researchers on a public website? |  | 🗸  Line 123 |  |
| 3. | Is code used for the study available on a public website to allow for reproduction or analysis of data |  | 🗸  Line 123 |  |
| DESIGN AND ANALYSIS: | | N/A | YES | NO |
| 4. | Was ethics approval obtained? |  | 🗸  Line 139 |  |
| 5a.  5b. | Was the sample size based on a formal sample size calculation done prior to starting the study?  If so, was the planned sample size adhered to? |  | 🗸  Line 134 |  |
|  |  |  | 🗸  Line 138 |  |
| 6. | Was data analysis blinded? |  | 🗸  Line 230 |  |
| REPORTING PRACTICES: | | N/A | YES | NO |
| 7. | Are any reporting guidelines specified (such as those found at www.equuator-network.org)? |  | 🗸  Line 119 |  |
| 8a.  8b.  8c. | All measures of variability defined in figures, tables and text?  Are any data summarized using standard error of the mean (SEM)?  If the SEM is used, are sample sizes specified for all reported SEM? |  | 🗸  Line 266-285 |  |
|  |  |  |  | 🗸 |
|  |  |  |  | 🗸 |
| 9a.  9b. | Were any data excluded?  If so, was a criterion given? |  |  | 🗸 |
|  |  |  |  | 🗸 |
| 10a.  10b. | If null-hypothesis testing of significance was used, is a probability threshold specified for all statistical tests?  If used, are exact probability values used throughout the report, excluding figure legends? |  | 🗸  Line 263 |  |
|  |  |  | 🗸  Line 266-285 |  |
| 11. | Are claims made for the importance and significance of results associated with a P-value grater than or equal to 0.05 (or other threshold) i.e. misleading spin of reported results? |  | 🗸  Line 266-285 |  |

| **Appendix 3.** R code of the study. |
| --- |
| library(ggplot2)  library(gridExtra)  shapiro.test(landing_rom$yas)  shapiro.test(landing_rom$boy)  shapiro.test(landing_rom$kilo)  shapiro.test(landing_rom$deneyim)  shapiro.test(landing_rom$inis_skoru)  shapiro.test(landing_rom$bmi)  shapiro.test(landing_rom$ankle_rom)  mod <- lm(boy ~ ., data=landing_rom)  cooksd <- cooks.distance(mod)  plot(cooksd, pch="*", cex=2, main="Influential Obs by Cooks distance") # plot cook's distance  abline(h = 3*mean(cooksd, na.rm=T), col="red") # add cutoff line  text(x=1:length(cooksd)+1, y=cooksd, labels=ifelse(cooksd>3*mean(cooksd, na.rm=T),names(cooksd),""), col="red") # add labels  group_1 <- subset(landing_rom, gruplar == 1)  group_1_inis_skor <- group_1$inis_skoru  ankle_rom1 <- group_1$ankle_rom  cor.test(group_1_inis_skor, ankle_rom1, method = "spearman")  cor_estimate <- -0.6371528  p_value <- 1.693e-07  z_value <- qnorm(0.975) # 0.975, çift yönlü 0.025'lik kritik değer  n <- length(group_1_inis_skor)  SE <- 1 / sqrt(n - 3)  lower_bound <- cor_estimate - z_value * SE  upper_bound <- cor_estimate + z_value * SE  cat("95% Güven Aralığı:", lower_bound, " - ", upper_bound, "\n")  group_2 <- subset(landing_rom, gruplar == 2)  group_2_inis_skor <- group_2$inis_skoru  ankle_rom2 <- group_2$ankle_rom  cor.test(group_2_inis_skor, ankle_rom2, method = "spearman")  cor_estimate <- 0.2211984  p_value <- 0.1351  z_value <- qnorm(0.975) # 0.975, çift yönlü 0.025'lik kritik değer  n <- length(group_1_inis_skor)  SE <- 1 / sqrt(n - 3)  lower_bound <- cor_estimate - z_value * SE  upper_bound <- cor_estimate + z_value * SE  cat("95% Güven Aralığı:", lower_bound, " - ", upper_bound, "\n")  landing_1 <- ggplot(group_1, aes(x=group_1_inis_skor, y=ankle_rom1)) +  geom_point(color = "navyblue", fill= "red", alpha = 0.6, shape = 23, size = 2, stroke= 2)+  geom_smooth() +  theme(axis.line = element_line(colour = "black"),  axis.text= element_text(face = "bold", size = 10),  panel.grid.major = element_blank(),  panel.grid.minor = element_blank(),  panel.border = element_blank(),  panel.background = element_blank(),  legend.direction = "vertical",  legend.key.height = unit(0.3, 'cm'),  legend.background = element_rect(fill = "transparent", color = NA),  legend.position = c(0.90, 0.85)) +  scale_y_continuous(name = "Ankle Joint Range of Motion",  limits = c(20, 50), expand = c(0, 0)) +  scale_x_continuous(name = "Landing Score",  limits = c(2.8, 11.2), expand = c(0, 0)) +  annotate("text", x = 9, y = 48, label = "r = -0.63, p = 0.00", size= 4)+  annotate("text", x = 9, y = 46.5, label = "95% CI = -0.90 to -0.36", size= 4)  landing_1    landing_2 <- ggplot(group_2, aes(x=group_2_inis_skor, y=ankle_rom2)) +  geom_point(color = "black", fill= "green", alpha = 0.6, shape = 21, size = 3, stroke= 2)+  geom_smooth() +  theme(axis.line = element_line(colour = "black"),  axis.text= element_text(face = "bold", size = 10),  panel.grid.major = element_blank(),  panel.grid.minor = element_blank(),  panel.border = element_blank(),  panel.background = element_blank(),  legend.direction = "vertical",  legend.key.height = unit(0.3, 'cm'),  legend.background = element_rect(fill = "transparent", color = NA),  legend.position = c(0.90, 0.85)) +  scale_y_continuous(name = "Ankle Joint Range of Motion",  limits = c(20, 48), expand = c(0, 0)) +  scale_x_continuous(name = "Landing Score",  limits = c(2.8, 9.5), expand = c(0, 0)) +  annotate("text", x = 7, y = 46, label = "r = 0.22, p = 0.13", size= 4)+  annotate("text", x = 7, y = 44.5, label = "95% CI = -0.05 to 0.49", size= 4)  landing_2  landing_plot <- (landing_1 +landing_2)  landing_plot  ggsave(plot = landing_plot,  filename = "landing.png",  width = 10,  height = 5,  dpi = 1000)  library (quantreg)  summary(landing_rom)  hist(Landing_score, prob=TRUE, col = "blue", border = "black")  lines(density(Landing_score))  ols_regress(group_1_inis_skor ~ ankle_rom1, data = group_1)  OLS <- lm(group_1$group_1_inis_skor ~ group_1$ankle_rom1)  summary (OLS)  Qreg25=rq(group_1_inis_skor ~ ankle_rom1, tau=0.25)  summary(Qreg25)  Qreg50=rq(group_1_inis_skor ~ ankle_rom1, tau=0.50)  summary(Qreg50)  Qreg75=rq(group_1_inis_skor ~ ankle_rom1, tau=0.75)  summary(Qreg75)  Qreg90=rq(group_1_inis_skor ~ ankle_rom1, tau=0.90)  summary(Qreg90)  Qreg95=rq(group_1_inis_skor ~ ankle_rom1, tau=0.95)  summary(Qreg95)  QR=rq(group_1_inis_skor ~ ankle_rom1, tau=seq(0.2, 0.8, by=0.1))  sumQR=summary(QR)  plot(sumQR)  library(quantregGrowth)  o <-gcrq(group_1_inis_skor ~ ankle_rom1,  data = group_1, sample_n(100), tau=seq(.10,.25,.50,.75,.90,l=3))  # par(mfrow=c(1,2)) # for several plots  plot(o, legend=TRUE, conf.level = .95, shade=TRUE, lty = 1, lwd = 3, col = -1, res=TRUE)  library(olsrr)  group_1 <- subset(landing_rom, gruplar == 1)  group_1_inis_skor <- group_1$inis_skoru  ankle_rom1 <- group_1$ankle_rom  group_2 <- subset(landing_rom, gruplar == 2)  group_2_inis_skor <- group_2$inis_skoru  ankle_rom2 <- group_2$ankle_rom  lr <- lm(group_1_inis_skor ~ ankle_rom, data = group_1)  mr <- rq(group_1_inis_skor ~ ankle_rom, tau = 0.90)  lr2 <- lm(group_2_inis_skor ~ ankle_rom2, data = group_2)  mr2 <- rq(group_2_inis_skor ~ ankle_rom2, tau = 0.90)  AIC(lr, mr) # => the lower AIC the better  AIC(lr2, mr2) # => the lower AIC the better  outliers <- ols_plot_resid_lev(lr)  outliers  outliers2 <- ols_plot_resid_lev(lr2)  outliers2  outliers <- ols_plot_resid_lev(lr)  outliers2 <- ols_plot_resid_lev(lr2)  p1 <- outliers +  geom_point(aes(color = "Analysis 1"), size = 2, stroke= 2, shape= 8) +  scale_color_manual(values = c("Analysis 1" = "#F8766D")) +  labs(title = "(A)")  p1  # lr2 için outlier'ları kırmızıyla işaretle  p2 <- outliers2 +  geom_point(aes(color = "Analysis 2"), size = 2, stroke= 2, shape=6) +  scale_color_manual(values = c("Analysis 2" = "#c77CFF")) +  labs(title = "(B)")  p2  library(patchwork)  combined_plot<- p1 / p2  combined_plot  ggsave(plot = combined_plot,  filename = "landingplott.png",  width = 9,  height = 5,  dpi = 800)  library(performance)  lr <- lm(group_1_inis_skor ~ ankle_rom1, data = group_1)  check_heteroscedasticity(lr)  lr2 <- lm(group_2_inis_skor ~ ankle_rom2, data = group_2)  check_heteroscedasticity(lr2)  # AMATEUR  l <- lm(group_1_inis_skor ~ ankle_rom1, data = group_1)  q10 <- rq(group_1_inis_skor ~ ankle_rom1, tau = .10)  q25 <- rq(group_1_inis_skor ~ ankle_rom1, tau = .25)  q51 <- rq(group_1_inis_skor ~ ankle_rom1, tau = .51)  q75 <- rq(group_1_inis_skor ~ ankle_rom1, tau = .75)  q90 <- rq(group_1_inis_skor ~ ankle_rom1, tau = .90)  library(gtsummary)  tbl_merge(  tbls = list(  tbl_regression(l) %>% bold_p(),  tbl_regression(q10, se = "nid") %>% bold_p(),  tbl_regression(q25, se = "nid") %>% bold_p(),  tbl_regression(q51, se = "nid") %>% bold_p(),  tbl_regression(q75, se = "nid") %>% bold_p(),  tbl_regression(q90, se = "nid") %>% bold_p()  ),  tab_spanner = c("OLS", "QR 10%","QR 25", "QR 50%", "QR75", "QR 90%")  )  #ELİTE  l <- lm(group_2_inis_skor ~ ankle_rom2, data = group_2)  q10 <- rq(group_2_inis_skor ~ ankle_rom2, tau = .10)  q25 <- rq(group_2_inis_skor ~ ankle_rom2, tau = .25)  q51 <- rq(group_2_inis_skor ~ ankle_rom2, tau = .51)  q75 <- rq(group_2_inis_skor ~ ankle_rom2, tau = .75)  q90 <- rq(group_2_inis_skor ~ ankle_rom2, tau = .90)  library(gtsummary)  tbl_merge(  tbls = list(  tbl_regression(l) %>% bold_p(),  tbl_regression(q10, se = "nid") %>% bold_p(),  tbl_regression(q25, se = "nid") %>% bold_p(),  tbl_regression(q51, se = "nid") %>% bold_p(),  tbl_regression(q75, se = "nid") %>% bold_p(),  tbl_regression(q90, se = "nid") %>% bold_p()  ),  tab_spanner = c("OLS", "QR 10%","QR 25", "QR 50%", "QR75", "QR 90%")  ) |
